# The Endocannabinoid Peptide RVD-Hemopressin Is a TRPV1 Channel Blocker

**DOI:** 10.3390/biom14091134

**Published:** 2024-09-08

**Authors:** Constanza Suárez-Suárez, Sebastián González-Pérez, Valeria Márquez-Miranda, Ingrid Araya-Duran, Isabel Vidal-Beltrán, Sebastián Vergara, Ingrid Carvacho, Fernando Hinostroza

**Affiliations:** 1 Facultad de Ciencias Agrarias y Forestales, Universidad Católica del Maule, Talca 3460000, Chile; constanza.suarez@alu.ucm.cl (C.S.-S.); sebastian.gonzalez.04@alu.ucm.cl (S.G.-P.); 2 Center for Bioinformatics and Integrative Biology (CBIB), Universidad Andrés Bello, Santiago 8370146, Chile; valeria.marquez@unab.cl (V.M.-M.); ingrid.araya.duran@gmail.com (I.A.-D.); 3 Centro de Investigación de Estudios Avanzados del Maule (CIEAM), Vicerrectoría de Investigación y Postgrado, Universidad Católica del Maule, Talca 3460000, Chile; ividal@ucm.cl; 4 Departamento de Medicina Traslacional, Facultad de Medicina, Universidad Católica del Maule, Talca 3460000, Chile; sebadiantian@gmail.com (S.V.); icarvacho@ucm.cl (I.C.); 5 Centro de Investigación en Neuropsicología y Neurociencias Cognitivas, Facultad de Ciencias de la Salud, Universidad Católica del Maule, Talca 3460000, Chile; 6 Centro para la Investigación Traslacional en Neurofarmacología, Universidad de Valparaíso, Valparaíso 2340000, Chile

**Keywords:** RVD-hemopressin, TRPV1 channel, calcium imaging, patch-clamp electrophysiology, molecular dynamics simulations

## Abstract

Neurotransmission is critical for brain function, allowing neurons to communicate through neurotransmitters and neuropeptides. RVD-hemopressin (RVD-Hp), a novel peptide identified in noradrenergic neurons, modulates cannabinoid receptors CB1 and CB2. Unlike hemopressin (Hp), which induces anxiogenic behaviors via transient receptor potential vanilloid 1 (TRPV1) activation, RVD-Hp counteracts these effects, suggesting that it may block TRPV1. This study investigates RVD-Hp’s role as a TRPV1 channel blocker using HEK293 cells expressing TRPV1-GFP. Calcium imaging and patch-clamp recordings demonstrated that RVD-Hp reduces TRPV1-mediated calcium influx and TRPV1 ion currents. Molecular docking and dynamics simulations indicated that RVD-Hp interacts with TRPV1’s selectivity filter, forming stable hydrogen bonds and van der Waals contacts, thus preventing ion permeation. These findings highlight RVD-Hp’s potential as a therapeutic agent for conditions involving TRPV1 activation, such as pain and anxiety.

## 1. Introduction

Neurotransmission is a crucial mechanism to transmit information from one neuron to another, allowing the brain to integrate, process, and respond to incoming information. Neurons do not only release neurotransmitters, but also co-release neuropeptides under certain conditions. Among these neuropeptides, a novel peptide, RVD-hemopressin (RVDPVNFKLLSH, RVD-Hp), has recently been unearthed within noradrenergic neurons [1]. These RVD-Hp-containing neurons project to the cerebral cortex, hypothalamus, hippocampus, and spinal cord [2].

Functionally, RVD-Hp has been identified as a dual modulator, acting as a negative allosteric modulator of the cannabinoid receptor CB1 (CB1R) and a positive allosteric modulator of CB2 (CB2R) in the presence of 2-arachidonoylglycerol [3,4]. CB1R is expressed in the plasma membrane and mitochondria [5,6,7]. Consequently, RVD-Hp emerges as a promising candidate for therapeutic intervention in psychiatric disorders by modulating plasma membrane CB1R, highlighting its potential to modulate the endocannabinoid system.

The administration of hemopressin (Hp) has been shown to induce anxiogenic and depressive-like behaviors in rodent models. This effect is attributed to the activation of the transient receptor potential vanilloid 1 (TRPV1) channel, as evidenced by the mitigating impact of SB366791, a TRPV1 antagonist, on anxious behaviors [8]. Intriguingly, RVD-Hp administration counteracts the anxiety- and depressive-like behaviors induced by hemopressin [9,10], suggesting a potential blockade of the TRPV1 channel by RVD-Hp.

The transient receptor potential (TRP) channels family involves several subfamilies, including the vanilloid (TRPVs) group [11]. TRPV1, a member of the TRPV subfamily, is a non-selective cationic homotetramer channel. It is known to be activated by various stimuli, including temperature, capsaicin (the principal compound in chili peppers) [12], and endocannabinoids such as anandamide (AEA) and 2-arachidonoylglycerol (2-AG) [13,14,15,16]. The TRPV1 channel protein is found in the central (CNS) and peripheral nervous system (PNS). In the CNS, TRPV1 is expressed in the prefrontal cortex, hypothalamus, hippocampus, amygdala, brainstem, and laminae I and II of the dorsal horn of the spinal cord [17,18,19,20,21,22,23,24,25,26,27]. In the PNS, it is located in dorsal root ganglion neurons, peptidergic and non-peptidergic C fibers, the nodal ganglion, and the sympathetic ganglion [28,29,30]. Structurally, the TRPV1 channel comprises an intracellular ankyrin repeat domain followed by six transmembrane segments (S1–S6), with S5 and S6 forming the pore, and an intracellular TRP domain located in its C-terminal [31].

Physiologically, the TRPV1 channel is crucial in pain and temperature perception. The administration of capsazepine, a TRPV1 antagonist, reduces thermal hyperalgesia. Moreover, TRPV1 knock-out mice exhibit sensitivity to noxious temperatures and lack thermal hyperalgesia [12,32,33,34]. Beyond its role in pain modulation, TRPV1 activation is also implicated in regulating anxious behavior [8,35,36,37], as TRPV1-deficient mice demonstrate reduced anxiety-like behavior [38]. Additionally, TRPV1 channel expression is notably upregulated in various inflammatory conditions, including arthritis, osteoarthritis, chronic asthma, and non-erosive reflux disease. In these contexts, TRPV1 antagonists have been reported to attenuate the inflammatory response [39,40,41].

Several TRPV1 modulators have been developed to alleviate pain [42,43,44,45]. Nonetheless, these compounds are often associated with adverse effects, including the development of drug tolerance and impaired body-temperature regulation [46]. Therefore, new TRPV1 antagonists with reduced or no side effects, low toxicity, and high efficacy are needed in biomedicine.

In this study, we provide evidence supporting RVD-Hp as a TRPV1 channel blocker. Our preliminary data show its putative binding site, located in the extracellular region of the pore. RVD-Hp is predicted to interact with specific amino acids of the TRPV1, including I642, G643, and M644 of the channel’s selectivity filter. The interaction between RVD-Hp and TRPV1 involves mostly hydrogen bonds and Van der Waals forces. Our study opens new alternatives to modulate TRPV1 by endogenous peptides, supporting novel pathways to regulate pain and other physiological and pathological conditions.

## 2. Materials and Methods

### 2.1. Cell Culture and Transfection

Dr. Alejandro Maureira, Universidad Católica del Maule, Chile, generously provided HEK293 cells. The cells were cultured in T75 flasks (Corning Inc., Corning, NY, USA) in a humidified atmosphere at 37 °C with 5% CO_2_ in Dulbecco’s Modified Eagle Medium (DMEM) with high glucose (Cytiva, Marlborough, MA, USA), supplemented with 10% fetal bovine serum (Cytiva, Marlborough, MA, USA) and a mixture of 100 IU/mL penicillin and 100 µg/mL streptomycin (Corning Inc., Corning, NY, USA) in a CO2CELL incubator (MMM Group, Munich, Germany). This procedure was used to maintain the cells for all of the experiments.

HEK293 cells are extensively used in biological research because they are adaptable to a wide range of culture conditions, exhibit high transfection efficiency, are well characterized, and have been widely used in heterologous expression systems, including in the transfection and overexpression of the TRPV1 channel [13,14]. HEK293 cells were transfected with the human TRPV1 channel coupled to GFP in its C-terminus (TRPV1-GFP, catalog #: RG217653, Origene, Rockville, MD, USA). This TRPV1-GFP plasmid induces the expression of a fully functional TRPV1, as previously reported [47,48]. Transfections were conducted using Lipofectamine 3000 (Invitrogen, Waltham, MA, USA), according to the manufacturer’s instructions. Experimental protocols were performed 24 h post-transfection.

### 2.2. Immunofluorescence

Transfected HEK293 cells were fixed and permeabilized using 4% paraformaldehyde for 20 min and PBS/0.3% BSA- 0.3% Triton X-100 solution, respectively. The samples were blocked by incubation with PBS/1% BSA and 10% donkey serum for 1 h at room temperature (RT). A rabbit anti-TRPV1 antibody was used as the primary antibody and incubated overnight (1:200, Thermo Fischer, Waltham, MA, USA). Donkey anti-rabbit IgG conjugated with Alexa Fluor 568 (Abcam Inc., Waltham, MA, USA) was used as a secondary antibody. The antibodies were diluted in PBS/0.3% BSA and 1% donkey serum. PBS/0.3% BSA and 0.1% Tween20 solution were used to wash the samples. DNA staining was performed using 0.01 mg/mL Hoechst 33258 (Invitrogen, Waltham, MA, USA). All the samples were mounted using VectaShield mounting media (Vector Laboratories, Newark, CA, USA). As a control, the primary anti-TRPV1 antibody was excluded from the experimental protocol. Immunolabeling was observed using Leica Stellaris 5 spectral confocal microscopy (FONDEQUIP-ANID EQM200122).

### 2.3. Calcium Imaging

The intracellular calcium (Ca^2+^) concentration was measured using Fura Red-AM (Invitrogen, Waltham, MA, USA). Untransfected and TRPV1-GFP-transfected HEK293 cells were cultured in 15 mm round coverslips previously treated with 0.1 mg/mL poly-L-lysine (Gibco, Billings, MT, USA). Cells were incubated with 5 µM Fura Red-AM, 0.01% Pluronic F127, and DMEM high-glucose medium for 20 min at 37 °C and 5% CO_2_. Then, the cells were washed and cultured during the experiment with the extracellular solution containing (in mM) 140 NaCl, 5 KCl, 2 CaCl_2_, 1 MgCl_2_, 10 HEPES, and 6 glucose, with pH adjusted to 7.4 with NaOH [13]. Calcium images were obtained every 3 s for 30 min using Leica Stellaris 5 spectral confocal microscopy. Cells containing Fura Red-AM were excited at 405 and 488 nm to stimulate Fura Red-AM bound and unbound to Ca^2+^, respectively. Fura Red emissions in bound and unbound states were acquired around their peaks at 610 nm and 670 nm, respectively. The bound and unbound fluorescence of Fura Red-AM was acquired using Leica LAS X v.4.3.0.24308 software. The Fura Red-Am bound and unbound ratio was calculated (405 nm/488 nm) and plotted. We used capsaicin (nº 92350, Cayman Chemicals, Ann Arbor, MI, USA), RVD-Hp (Synbio Technologies, Monmouth Junction, NJ, USA), and the TRPV1 channel antagonist SB366791 (nº 11019, Cayman Chemicas, Ann Arbor, MI, USA). Untransfected cells and incubation with dimethyl sulfoxide (DMSO, Cell Signaling Technology, Danvers, MA, USA) were used as controls. Imaging was performed at room temperature.

### 2.4. Patch-Clamp Electrophysiology

Untransfected and transfected HEK293 cells with the human TRPV1-GFP were recorded using a whole-cell voltage clamp. Cells were cultured in 15 mm round coverslips pretreated with 0.1 mg/mL poly-L-lysine (Gibco, Billings, MT, USA) and transfected with TRPV1-GFP plasmid. TRPV1-GFP-positive cells were identified using a 405 nm LED light in a Nikon Eclipse Ts2 inverted microscope. Borosilicate pipettes (nº 1B150F-4, World Precision Instruments, Sarasota, FL, USA) of 2.8’5 MΩ were used. Pipettes were pulled with a P-97 Micropipette Puller (Sutter Instruments, Novato, CA, USA). The extracellular solution contained (in mM) 140 NaCl, 5 KCl, 2 CaCl_2_, 1 MgCl_2_, 10 HEPES, and 6 glucose at pH 7.4. The internal solution contained (in mM) 140 CsCl, 4 MgCl_2_, 10 EGTA, and 10 HEPES-CsOH at pH 7.3. All the experiments were recorded using a HEKA EPC9 amplifier. The holding potential was −80 mV. A 500 ms voltage ramp from −80 to 80 mV was applied to record TRPV1 currents. The sample frequency was 50 kHz, and the signals were filtered at 2.9 kHz using PatchMaster software. Data were analyzed using Clampfit v.7 software (Molecular Devices, San Jose, CA, USA). The extracellular solution and ligands were administered using a VC-8 perfusion system (~2 mL/s, Warner Instruments, Holliston, MA, USA). We used capsaicin (Cayman Chemicals, nº 92350), SB366791 (nº 11019, Cayman Chemicals, Ann Arbor, MI, USA), and RVD-Hp (Synbio Technologies, Monmouth Junction, NJ, USA). We used dimethyl sulfoxide (DMSO, Sigma-Aldrich, St. Louis, MO, USA) and untransfected cells as controls. Electrophysiological recordings were performed at room temperature.

### 2.5. Cell-Penetrating Peptide Analysis

An RVD-Hp sequence was used as input to predict the probability of the peptide being a cell-penetrating peptide using BChemRF-CPPred (Beyond Chemical Rules-based Framework for CPP prediction) [49].

### 2.6. Molecular Docking

We used the TRPV1 channel structure in the open configuration (PDB ID: 7LPE [50]). The missing regions (111-205, 270-281, and 603-625) were completed using the AlphaFold 2 predicted structure for the TRPV1 channel (ID: O35433). The RVD-hemopressin (RVD-Hp) peptide was obtained from the alpha chain hemoglobin (PDB ID: 1O1M). The completed and relaxed structure of the TRPV1 channel was used to dock the RVD-hemopressin peptide using HADDOCK2.4 [51]. The complex with the best HADDOCK score was used to perform a molecular dynamics simulation.

We also docked capsaicin (Cap) to the TRPV1 channel in the vanilloid pocket using AutoDock Vina software v.1.2.3 [52]. The complex with the best energy was selected to perform molecular dynamics (MD) simulations.

### 2.7. Molecular Dynamics Simulations

Each system was built using the CHARMM-GUI web server [53,54,55]. The TRPV1-Cap and TRPV1-Cap-RVD-Hp complexes were inserted into a phosphatidylcholine membrane bilayer (POPC), solvated using the OPC water model and ionized with 150 mM KCl.

Systems were prepared considering ff19SB [56], Lipid17 [57], GAFF, and OPC force fields for proteins, the membrane bilayer, capsaicin, and water and ion molecules. The systems were minimized and equilibrated using an NVT ensemble and the CHARMM-GUI suggested protocol for membrane proteins. Data production simulations of 1 µs were performed using an NPT ensemble in both systems. We used the AMBER22 suite to run and analyze the simulations [58]. The temperature was kept constant using the velocity rescale (v-rescale) thermostat [59]. The semi-isotropic Berendsen barostat [60] was used to keep the pressure at 1 atm, 310 K, and 1 bar.

After the equilibration, systems were subjected to an external electric field along the *z*-axis to induce the membrane voltage of −300 mV. The voltage (V) was calculated with

V = ELz,

where E represents the applied electric field and Lz is the length of the simulation box along the *z*-axis, as reported previously [61,62].

Analysis regarding ion occupancy and translocation was carried out using the MDAnalysis package v2.4.0 [63].

### 2.8. Contact Area

To determine the contact area of RVD-Hp to the TRPV1 channel, we built an in-house Tcl script that quantifies the solvent-accessible surface area (SASA) of the ligand and complex. The contact area was calculated using the following equation:

Contact Area = SASAligand − (SASAcomplex − SASAligand)

### 2.9. Protein–Ligand Contacts

Contact types and frequency were obtained using the GetContacts application (https://getcontacts.github.io/, accessed on 21 June 2024). The hydrogen bond interaction was calculated using a donor–acceptor distance < 3.5 Å and an angle of 180°–70°. Van der Waals interactions were computed using the following equation:

|AB| < Rvdw(A) + Rvdw(B) + 0.5

The flare plot was generated using the MD contacts website https://gpcrviz.github.io/flareplot/?p=create (accessed on 21 June 2024).

### 2.10. Binding Free Energy

The binding free energy calculation was performed throughout the trajectory at intervals of 100 ns using the Molecular Mechanics—Generalized Born Surface Area (MM-GBSA) method from the AMBER22 suit [58]. MM-GBSA aims to provide insights into binding thermodynamics by combining molecular mechanics calculations to model the protein–ligand complex with a continuum solvent model to account for solvation effects.

### 2.11. Structure Visualization

The figures were obtained using Visual Molecular Dynamics (VMD) software v1.9.3 [64].

### 2.12. Pore Radius Calculation

The radius of the TRPV1 channel pore was measured at 1 µs of simulation using the Hole Program v.2.2.005 [65].

### 2.13. Root Mean Square Deviation

The root mean square deviation (RMSD) was calculated using the RMSD Trajectory Tool plugin of the VMD software.

### 2.14. Radius of Gyration

The radius of gyration was measured using a built-in-house Tcl script.

### 2.15. Root Mean Square Inner Product

The root mean square product (RMSIP) [66] was calculated using bio3d package v2.4-4 of R software (http://thegrantlab.org/bio3d/, accessed on 26 August 2024). RStudio v.1.4.1103 was used for this analysis.

### 2.16. Statistical Analysis

The data were analyzed and plotted using GraphPad software v10.0.2. We used the Shapiro–Wilk test to determine the data distribution. Since the data did not exhibit a normal distribution, we used the Mann–Whitney test for two-group comparisons and the Kruskal–Wallis test for non-parametric data comparisons of more than two groups. Statistically significant values were considered when *p* values were * *p* < 0.05, ** *p* < 0.01, *** *p* < 0.001, and **** *p* < 0.0001.

## 3. Results

### 3.1. RVD-Hemopressin Reduces the Ca^2+^ Influx through the TRPV1 Channel in HEK293 Cells

**Figure 1:**
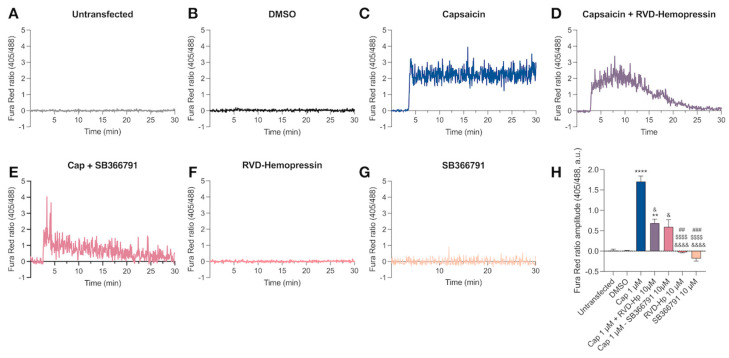
RVD-Hp reduces the calcium influx in HEK293-transfected cells. Representative traces of the Fura red ratio (405 nm/488 nm) in (**A**) HEK293-untransfected cells (n: 26) and -transfected cells after (**B**) DMSO (n: 20), (**C**) 1 µM cap (n: 58), (**D**), 1 µM cap + 10 µM RVD-Hp (n: 60), (**E**) 1µM cap + 10 µM SB366791 (n: 20), (**F**) 10 µM RVD-Hp (n: 59), and (**G**) 10 µM SB366791 (n: 51) administration. (**H**) Mean amplitude of Fura Red ratio (405 nm/488 nm) calculated in the last minute of the experiment (mean ± SEM). *: Comparison to DMSO, ** *p* < 0.01, **** *p* < 0.0001. &: Comparison to Cap, & *p* < 0.05, &&&& *p* < 0.0001. $: Comparison to Cap + RVD-Hp, $$$$ *p* < 0.0001. #: Comparison to Cap + SB366791, ## *p* < 0.01, ### *p* < 0.001.

To investigate the effect of RVD-Hp on the TRPV1 channel, we transfected HEK293 cells with the human TRPV1 channel coupled to the green fluorescent protein (hTRPV1-GFP). The immunolabeling with the anti-TRPV1 channel antibody showed that only transfected cells were expressed the channel (Figure S1). Then, we performed Ca^2+^ imaging to evaluate the hTRPV1-GFP response to RVD-Hp using the fluorescent Ca^2+^ sensor Fura Red-AM. As a control, we used the agonist of TRPV1, capsaicin (Cap, 1 µM). Cap did not induce an increase in intracellular Ca^2+^ concentration in untransfected cells (Figure 1A,H). Similarly, DMSO administration in hTRPV1-GFP-positive cells did not increase the Fura Red-AM ratio (Figure 1B,H). Cap produced an increase in intracellular Ca^2+^ concentration (Figure 1C,H, **** *p* < 0.0001) that was partially blocked by the administration of 10 µM of RVD-Hp (Figure 1D, H, * *p* < 0.032) or 10 µM of SB366791 (Figure 1E,H, * *p* < 0.0113). Neither 10 µM RVD-Hp nor 10 µM SB366791 augmented the intracellular Ca^2+^ (Figure 1F,G). Our data show that RVD-Hp blocks the Ca^2+^ influx mediated by TRPV1 in HEK293 cells.

### 3.2. RVD-Hp Is an TRPV1 Channel Antagonist

**Figure 2:**
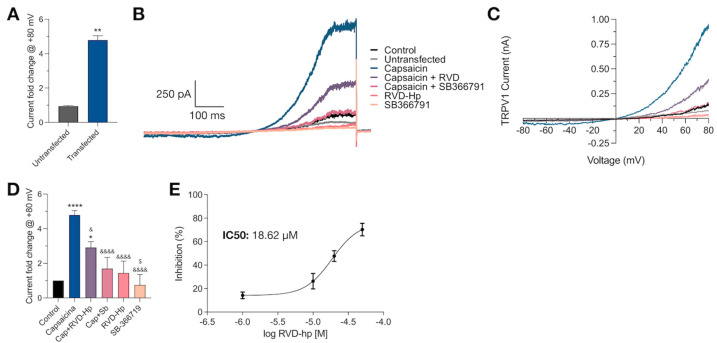
RVD-Hp is a TRPV1 channel blocker. (**A**) Comparison of current fold change measured at +80 mV in response to Cap (1 µM). (**B**) Representative traces of TRPV1 channel currents in response to a voltage ramp in the presence of 1 µM Cap (blue line), 1 µM Cap + 10 µM RVD-Hp (purple line), 1 µM Cap + 10 µM SB366791 (dark pink line), 10 µM RVD-Hp (pink line), and 10 µM SB36671 (yellow line). (**C**) Representative current (nA) versus voltage (mV) plot for all tested conditions. (**D**) Average outward current fold change amplitude at +80 mV (Control n: 7; Cap n: 7; Cap + RVD-Hp n: 4; Cap + SB366791 n: 4; RVD-Hp n: 4; SB366791 n: 3). *: Comparison to control, * *p* < 0.0186. ** *p* < 0.01. **** *p* < 0.0001. &: Comparison to capsaicin. & *p* < 0.0214, &&&& *p* < 0.0001. $: Comparison to capsaicin + RVD-Hp. $ *p* < 0.0323. (**E**) Average concentration–response curve for RVD-Hp (1, 10, 20, and 50 µM, mean ± SEM).

To further explore the blockade of the TRPV1 channel by RVD-Hp, we carried out voltage patch-clamp recordings in whole-cell configuration. We did not observe TRPV1 currents in response to a voltage ramp protocol in untransfected cells after administering 1 µM Cap (Figure 2A). In contrast, HEK293 cells expressing the TRPV1 channel showed Cap-induced currents (**** *p* < 0.0001) that were partially blocked by 10 µM RVD-Hp (* *p* < 0.0214) and blocked by 10 µM SB366791 (**** *p* < 0.0001), a TRPV1 channel antagonist. Neither 10 µM RVD-Hp nor 10 µM SB366791 alone induced TRPV1 currents; they were significantly different from the Cap-induced TRPV1 current (***** *p* < 0.0001) and were not different from the control (Figure 2B–D). Also, the administration of varying RVD-Hp concentrations (1, 10, 20, and 50 µM) revealed that the blockade of TRPV1 current follows a concentration-dependent relation (Figure 2E). The TRPV1 channel inhibition by RVD-Hp has an IC50 value of 18.62 µM (Figure 2E). Thus, our data indicate that the endocannabinoid peptide RVD-Hp is a TRPV1 channel blocker.

### 3.3. Molecular Simulations of RVD-Hp and TRPV1 Show an Interaction between RVD-Hp and the Selectivity Filter of the TRPV1 Channel

**Figure 3:**
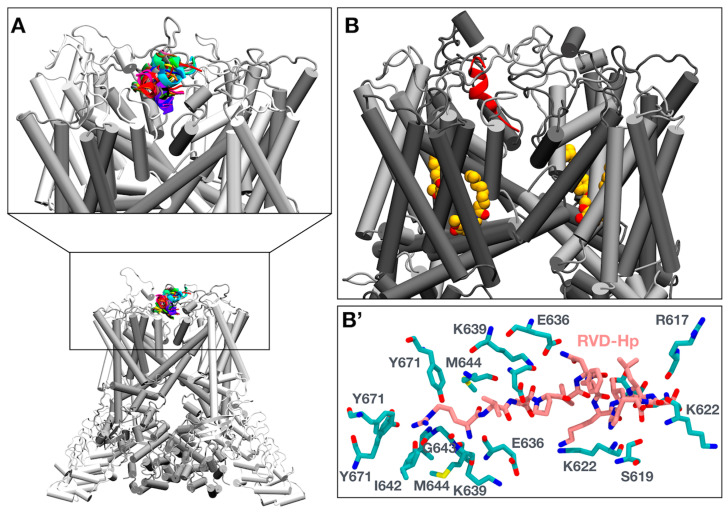
RVD-Hp interacts with the selectivity filter of the TRPV1 channel. (**A**) TRPV1-RVD-Hp docking results using HADDOCK2.4. (**B**) RVD-Hp (red)–TRPV1 channel (gray)–capsaicin (yellow) interaction at 1 µs of simulation. (**B’**) RVD-Hp (pink) contacts amino acids forming the TRPV1 channel selectivity filter.

To find a putative binding site of RVD-Hp in the TRPV1 channel, we assessed whether RVD-Hp could be a cell-penetrating peptide using BChemRF-CPPred [49]. With an 82.21% probability, RVD-Hp is not a cell-penetrating peptide. Thus, the binding site could be located in the extracellular region of the TRPV1 channel. Then, we performed a protein–peptide docking using the HADDOCK2.4 web server [51]. We obtained 32 different conformations located close to the pore of the TRPV1 channel (Figure 3A). We selected the model with the best HADDOCK score to carry out a 1 µs MD simulation. The root mean square deviation (RMSD), radius of gyration, and root mean square inner product (RMSIP) revealed that both complexes were stable (Figure S2). We observed that RVD-Hp locates the R1 of its N-terminal region in the TRPV1 channel pore (Figure 3B), where it interacts with I642, G643, and M644, amino acids that constitute the selectivity filter of the TRPV1 channel (Figure 3B’). Capsaicin interacts with Y511, L515, M547, T550, L553, E570, and I573 (Figure S3).

**Figure 4:**
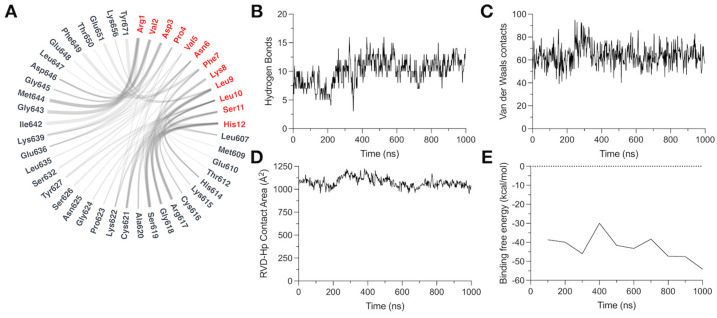
TRPV1 channel–RVD-Hp molecular interaction. (**A**) Flare plot showing all the amino acids involved in the RVD-Hp binding. Thicker lines represent contacts present for a longer period of time in the simulation. Peptide amino acids are colored in red. The number of (**B**) hydrogen bonds and (**C**) Van der Waals contacts throughout the simulation. (**D**) Contact area of interaction. (**E**) Binding free energy of the TRPV1 channel–RVD-Hp interaction.

The flare plot illustrates the amino acids involved in the TRPV1–peptide interaction. RVD-Hp R1 interacts with I642, G643, M644, and Y671; V2 interacts with G643, M644, G645, and D646; and D3 interacts with K639 and M644 (Figure 4A). On the other hand, in the C-terminal region of RVD-Hp, L10 interacts with C616, G618, and Y627; S11 interacts with R617, G618, and S629; and H12 interacts with R617 and S619 (Figure 4A). Then, we assessed and calculated the interaction types to further characterize the TRPV1 channel–RVD-Hp interaction. We found that hydrogen bonds and Van der Waals contacts mainly mediate the binding. RVD-Hp formed, on average, 10 hydrogen bonds (Figure 4B) and 64 Van der Waals contacts with the TRPV1 channel throughout the simulation (Figure 4C). The average contact area of TRPV1-RVD-Hp binding was 1076 Å2 (Figure 4D) with an average binding free energy of −42.6 kcal/mol (Figure 4E).

### 3.4. Molecular Dynamics Show That RVD-Hp Prevents Ion Permeation

**Figure 5:**
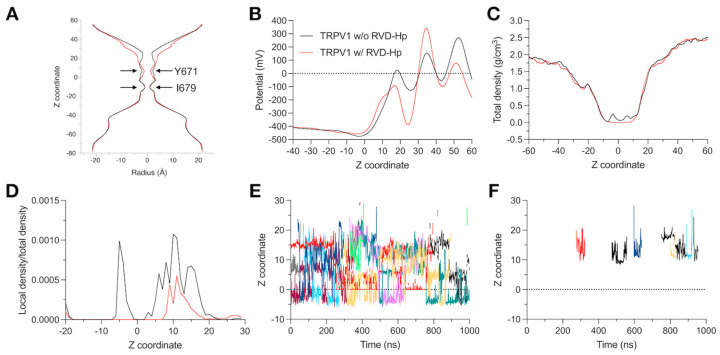
RVD-Hp prevents ion permeation. (**A**) Pore radius at 1 µs of simulation. (**B**) Potential through the pore. (**C**) Ion total density and (**D**) local density/total density in the pore. Ions present close to the pore in the TRPV1 channel (**E**) without and (**F**) with RVD-Hp. (**E**,**F**) Each line represents a different K^+^ ion. Black line: TRPV1 without RVD-Hp. Red line: TRPV1 with RVD-Hp.

Both systems, TRPV1 w/RVD-Hp and TRPV1 w/o RVD-Hp were subjected to a simulation using an electric field to replicate experimental conditions. After 1 µs of simulation, we evaluated whether RVD-Hp induces pore closure by measuring the pore radius of the channel at the end of the simulation using HOLE software [65]. Our findings indicate that RVD-Hp induces a more significant narrowing of the TRPV1 pore than the system without RVD-Hp at Y671 (Figure 5A).

Additionally, RVD-Hp alters the electric potential along the pore of the TRPV1 channel. TRPV1 without RVD-Hp shows positive potential peaks in the selectivity filter region and the extracellular region of the pore (z~30 Å), consistent with potassium (K^+^) binding sites. In contrast, the TRPV1 with RVD-Hp system first shows a pronounced peak at the entrance (z~30 Å), which becomes strongly negative when entering the selectivity filter (z~20 Å, Figure 5B). This indicates that the presence of the peptide significantly changes the electrostatic potential of the region. To determine if this change implies a blockage in ion translocation along the channel, we estimated the total and local density of K^+^ ions along the TRPV1 pore. We observed that the total and local densities of K^+^ ions decreased in the presence of RVD-Hp (Figure 5C,D).

While in TRPV1 without the RVD-Hp system, K^+^ occupancy is verified in the selectivity filter (0 Å < z < 20 Å) and the cavity towards the intracellular side of the filter (z~−5 Å), in TRPV1 with the RVD-Hp system, only a smaller peak in density can be observed in the filter region (z~10 Å, Figure 5D).

We obtained the trajectory along the *z*-axis of the K^+^ ions visiting the filter. About 10 ions visit the channel filter without RVD-Hp, residing in the area for up to hundreds of nanoseconds. In contrast, K^+^ ions visit TRPV1 with RVD-Hp only three times, with their residence times in the filter being just a few tens of nanoseconds (Figure 5E,F). All these findings are consistent with the ion density graphs and support our data that RVD-Hp blocks the TRPV1 channel pore and prevents ion permeation.

## 4. Discussion

Cannabinoid peptides, such as RVD-Hp, have emerged as promising therapeutic molecules due to their ability to modulate the endocannabinoid system. However, their interactions with other receptor targets still need to be explored. We used Ca^2+^ imaging and patch-clamp recordings to demonstrate that RVD-Hp is a TRPV1 channel blocker. Furthermore, molecular docking and MD simulations suggest that RVD-Hp binds to the extracellular side of the TRPV1 channel, specifically interacting with amino acids in the selectivity filter and preventing K^+^ occupancy of the selectivity filter and ion permeation across the channel.

The TRPV1 channel exhibits complex pharmacology modulated by temperature [12], endocannabinoids [13,14,15,16], peptides [67], protons [68], and cytokines [69,70], and regulated by kinases [71,72]. The TRPV1 channel is expressed in the medial prefrontal cortex, hippocampus, striatum, hypothalamus, periaqueductal gray, and substantia nigra [73,74,75,76,77]. Its activation by endocannabinoids like AEA and 2-AG in the brain is critical for synaptic transmission and plasticity in these regions [78,79,80]. TRPV1 channel activation in excitatory presynaptic neurons increases the spontaneous excitatory postsynaptic current frequency [73]. In the postsynaptic neuron, the TRPV1 channel mediates long-term depression [78,79,80]. Noradrenergic neurons, which project to the cortex, hippocampus, and periaqueductal gray, contain RVD-Hp [2]. RVD-Hp has also been detected in solutions maintaining brain slices, suggesting its release from noradrenaline-releasing neurons [81]. Thus, noradrenergic neurons might modulate TRPV1-mediated neurotransmission by releasing RVD-Hp, adding complexity to synaptic transmission processes.

Molecular dynamics (MD) simulations allow studying protein dynamics at an atomic level under physiological conditions, enhancing our understanding of various proteins, including ion channels like TRPV1 [82]. These simulations provide insight into their function, conformational changes, and interactions with different ligands [68,83,84,85,86,87]. Our molecular docking and dynamics simulations indicate that RVD-Hp blocks the TRPV1 channel via the extracellular side, preventing ion flow without inducing channel closure. This mechanism differs from other TRPV1 antagonists like capsazepine and SB366791 [88,89], which bind to the vanilloid pocket and compete with capsaicin, requiring four molecules to induce channel closure. In contrast, our model predicts that a single RVD-Hp molecule can block ion permeation. Phosphoinositide and phosphatidylinositol 4,5-biphosphate also favor the close state of the TRPV1 channel by binding the vanilloid binding pocket. In contrast, lysophosphatidic acid interaction with the vanilloid pocket induces channel opening by repositioning the Y671 within the pore [90]. According to our model, a single RVD-Hp molecule can obstruct ion permeation and reposition Y671 to narrow the pore. In addition, RVD-Hp does not exhibit characteristics of a cell-penetrating peptide; therefore, its mechanism of action could likely be extracellular and not in the transmembrane region of the channel. Further experiments are needed to confirm the exact binding site of RVD-Hp on the TRPV1 channel.

Hp peptide administration induces anxiety-like behavior in rats [8,9], an effect that is mitigated by the TRPV1 channel blocker SB366791 [8]. Despite this, evidence of a direct interaction between Hp and the TRPV1 channel is lacking. We speculate that Hp binds and activates TRPV1, possibly engaging the same binding site as RVD-Hp. Notably, Hp lacks the arginine that interacts with the channel’s selectivity filter, suggesting that it may not block TRPV1. In agreement with this, the neuropeptide oxytocin has been shown to activate the TRPV1 channel and modulate nociception by interacting with K603, N628, Y631, S632, L635, F649, T650, and K656, located in the extracellular side of the channel [67]. Similarly, RVD-Hp interacts with TRPV1 through S632, L635, F649, and K656. Therefore, Hp could bind to the same binding site as oxytocin to activate the TRPV1 channel.

The structural insights on TRPV1 described in the present study contribute to understanding the structural and functional aspects of the channel, aiding in the design of more effective and safer drugs. For example, new peptidic inhibitors can be designed based on RVD-Hp to be more selective, potentially reducing side effects such as hyperthermia, a significant issue with small-molecule TRPV1 inhibitors. Moreover, peptidic inhibitors can be engineered to bind specific sites on TRPV1, such as the outer pore region, which can enhance their specificity and reduce off-target effects. Additionally, peptidic inhibitors can be combined with other treatments to enhance their efficacy, such as low doses of capsaicin for prolonged analgesic effects [91].

Since the TRPV1 channel plays a role in pain and anxiety, antagonists and blockers of this channel might be used to treat these conditions. Thus, RVD-Hp emerges and holds potential as a novel modulator for non-opioid analgesia and anxiety treatment. The intracranial administration of RVD-Hp has been shown to prevent anxiety- and depressive-like behavior in rats, supporting its therapeutic potential [9]. Moreover, since RVD-Hp effectively blocks the open TRPV1 channel, it presents a promising approach to inhibit the population of TRPV1 channels that are pathologically over-activated, thereby potentially reducing side effects. This contrasts with competitive antagonists such as capsazepine and SB366791 [88,89], which prevent channel opening by displacing the agonist from their binding site. Future studies should focus on the clinical application of RVD-Hp in treating anxiety and pain, leveraging its unique mechanism of TRPV1 channel modulation.

Capsaicin creams and patches have been used in the treatment of muscle pain, neuropathic pain, arthritis, and migraines [92,93,94,95,96]. Nonetheless, Cap exhibits limited efficacy and bioavailability in vivo, and poor water solubility [97]. Furthermore, the oral administration of Cap can lead to side effects such as irritation, orofacial pain, and gastric ulcers [98]. CNTX-4975 has been developed as a treatment option for pain in individuals with osteoarthritis. However, this compound produces adverse effects, including nausea, hypotension, oral hypoesthesia, and erythema [99]. TRPV1 channel antagonists have also been developed to treat pain, but some of them increase body temperature [46,100], while others decrease it [101]. Similarly, TRPV1 channel blockers have been tested for anxiety treatment, but they also increase body temperature [102]. RVD-Hp emerges as a good candidate since it is an endogenous peptide, although whether RVD-Hp administration alters body temperature remains elusive.

Chronic pain is often accompanied by the development of depression [103]. RVD-Hp has been shown to reduce anxiety-like behavior in rats [9], suggesting its potential use in treating individuals suffering from both pain and depression. However, a significant challenge in utilizing RVD-Hp as a therapeutic agent lies in the inherent sensitivity of peptides to enzymatic degradation [104,105]. Specifically, RVD-Hp exhibits low stability in plasma and cannot cross the blood–brain barrier [106]. Developing carriers that can stabilize RVD-Hp and facilitate its delivery to the brain is essential to overcome these limitations. Additionally, evaluating RVD-Hp’s toxicity and bioavailability is critical before considering future clinical trials.

## 5. Conclusions

This study provides compelling evidence that RVD-Hp is an effective TRPV1 channel antagonist, capable of reducing Ca^2+^ influx and blocking ion currents through TRPV1 channels. The detailed interaction mapping between RVD-Hp and TRPV1 reveals specific binding sites and interaction types, offering valuable insights into the mechanism of TRPV1 inhibition by endocannabinoid peptides. These findings contribute to understanding TRPV1 modulation and have potential implications for developing new analgesic and anti-inflammatory therapies targeting TRPV1 channels.

## Data Availability

Data are available upon request to the corresponding author.

## Supplementary Materials

The following supporting information can be downloaded at: https://www.mdpi.com/article/10.3390/biom14091134/s1, Figure S1: The TRPV1 channel is present only in HEK293-transfected cells; Figure S2: Capsaicin interaction with the TRPV1 channel; Figure S3: Capsaicin interaction with the TRPV1 channel. The TRPV1 amino acids (cyan) contact with capsaicin (yellow) throughout the simulation.
