# Supplementary material for: The Endocannabinoid Peptide RVD-Hemopressin Is a TRPV1 Channel Blocker
Source: Biomolecules. 2024 Sep 8;14(9):1134. doi: 10.3390/biom14091134 (PMC11430712; doi:10.3390/biom14091134)
Supplement: Supplementary file 1 [file biomolecules-14-01134-s001.zip › biomolecules-3162161-supplementary.pdf]

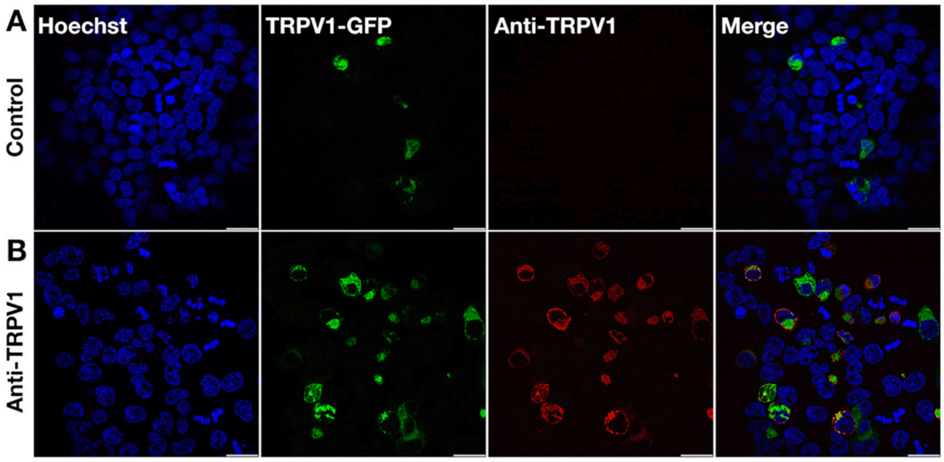

Figure S1: *The TRPV1 channel is present only in HEK293 transfected cells*. Immunofluorescence of HEK293 transfected cells (**A**) without and (**B**) with the anti-TRPV1 channel antibody incubation. Scale: 50 µm.

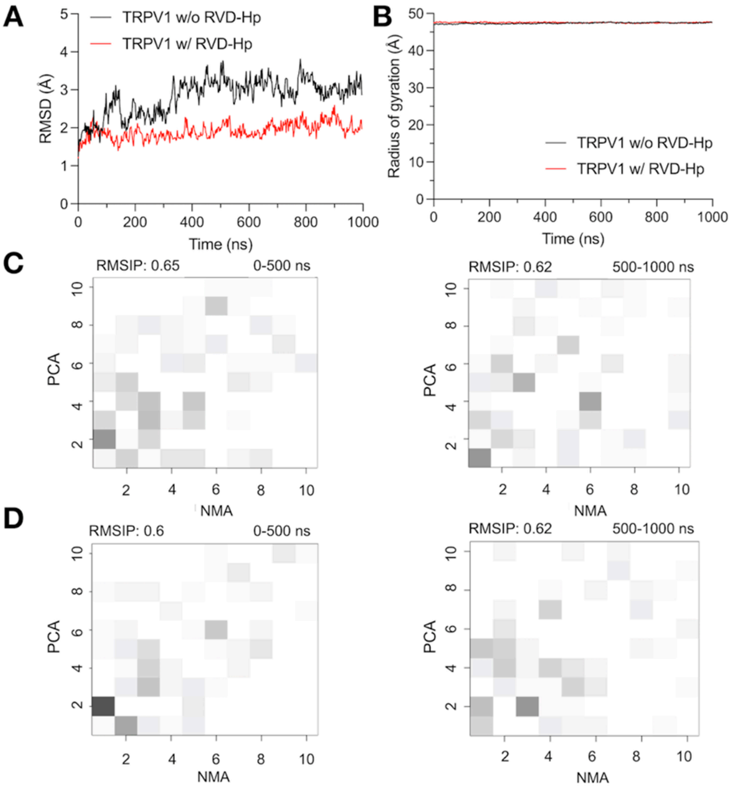

Figure S2: *Stability evaluation*. **A.** Root mean square deviation (RMSD) and (**B**) radius of gyration of the TRPV1 channel with and without RVD-Hp. Root mean square inner product (RMSIP) of the first (left) and last (right) 500 ns of (**C**) the TRPV1 channel without and (**D**) with RVD-Hp. PCA: Principal component analysis. NMA: Normal mode analysis.

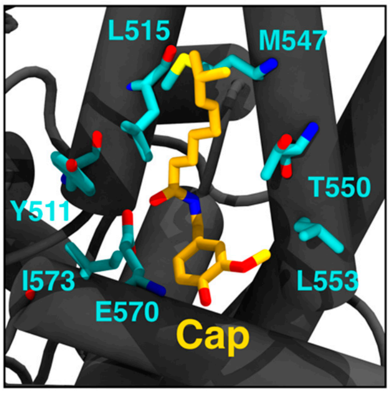

Figure S3: *Capsaicin interaction with the TRPV1 channel.* The TRPV1 amino acids (cyan) contact with capsaicin (yellow) throughout the simulation.
